# Global nexus of smoking prevalence, healthcare quality and respiratory cancer mortality: a cross-continental study

**DOI:** 10.1186/s12913-025-13508-9

**Published:** 2025-10-06

**Authors:** Lakindu Piumika, Disuri Silva, Roshinie De Silva, Ruwan Jayathilaka, Colinie Wickramaarachchi, Lochana Rajamanthri

**Affiliations:** 1 SLIIT Business School, Sri Lanka Institute of Information Technology, New Kandy Road, Malabe, Sri Lanka; 2 Department of Information Management, SLIIT Business School, Sri Lanka Institute of Information Technology, New Kandy Road, Malabe, Sri Lanka; 3 Department of Business Management, SLIIT Business School, Sri Lanka Institute of Information Technology, New Kandy Road, Malabe, Sri Lanka

**Keywords:** Healthcare access and quality index, Smoking prevalence, Lung neoplasms, Bronchitis, Tracheal neoplasms, Two-way ANOVA

## Abstract

**Background:**

Smoking causes Trachea, Bronchus, and Lung Cancer (TBLC) mortality, depicting a strong correlation, while the quality of healthcare access in countries considerably impacts health outcomes. This study evaluates the differential effect in the interplay between Smoking Prevalence (SP) and health care, employing the Healthcare Access and Quality (HAQ) index towards the TBLC mortality rates across diverse continents and globally.

**Methods:**

The data covering a 30-year period for 204 countries globally was categorised based on the level of SP (Low, Moderate, High, Critical) and the quality of healthcare access (Poor, Limited, Adequate, Optimal). A two-way ANOVA was utilised to analyse the patterns and variations in TBLC mortality rates across these categories, exploring the interactions between SP and the HAQ Index.

**Results:**

Distinct patterns were observed in each continent, highlighting the complex interactions between the HAQ Index and SP, which lead to varying health outcomes. The results indicate that regions with an optimal HAQ Index and low SP have lower TBLC death rates, whereas those with a poor HAQ Index and critical SP exhibit higher death rates.

**Conclusion:**

The findings emphasise the need to address both smoking prevalence and healthcare facilities globally. By improving healthcare access and reducing smoking rates, governments can significantly lower TBLC mortality rates. This study underlines the importance of integrating public health policies that limit smoking prevalence with enhancements in healthcare systems to promote better health and well-being.

**Supplementary information:**

The online version contains supplementary material available at 10.1186/s12913-025-13508-9.

## Background

Lung cancer is a dominant cancer-related mortality profile worldwide, and smoking is the most significant risk factor that advances this lethal disease. Smoking and Lung Cancer (LC) has a strong association where smokers have a higher risk than non-smokers [1]. High Smoking Prevalence (SP) is firmly correlated with Trachea, Bronchus, and Lung Cancer (TBLC), where a greater risk of evolving LC mortality rates occurs parallelly with the increase of individual smokers within a population and the increase of cigarettes consumed per day [2]. SP reveals the population percentage that currently smokes and has a smoking history [3–5]. These leads to cellular damage and cancer cell development mainly through entering harmful substances such as carcinogens and toxins into the lungs. TBLC, on the contrary, depicts the deaths in each population caused due to LC expressed as a rate per 100,000 people [6–8].

On the other hand, this devastating disease is variated regional-wise with high SP and healthcare access levels. The Healthcare Access and Quality (HAQ) index is utilised to quantify the healthcare and quality of the countries. The risk standardised death rates for 32 causes of death that should not recur in a quality and timely healthcare environment are further considered. This indicator is employed in research studies to access healthcare with the other considered variables (“Healthcare Access and Quality Index based on mortality from causes amenable to personal health care in 195 countries and territories, 1990–2015: a novel analysis from the Global Burden of Disease Study 2015,” [9; 10].

Further, the solid and well-documented nexus between TBLC, SP and the HAQ index based on various contributing factors such as healthcare systems, cultural norms, smoking patterns and behaviours, lung cancer remains the leading cause of mortality globally. Considering the Asian continent, countries like China and India reflect higher smoking rates which elevate the TBLC although variations occur between the countries in the continent [11–13]. Smoking prevalence rates within African continent countries differ tremendously. The TBLC are generally lower than the other continents [14, 15]. Shifting towards the American continent, South America historically, the SP rates are driven towards moderate to high, while TBLC has been significant and a leading cause in the North American continent parallelly [16, 17]. The LC mortality rates show a decline with time in specific countries as a result of the precautionary measures taken through the responsible authorities.

Europe portrays revealing variations of SP and TBLC in specific countries of the continent, where east European countries depict higher SP rates which have elevated the TBLC over the years with slight changes. On the contrary, western European countries show a decline in SP due to the preventive measures followed, while, as a consequence, the TBLC too shows a decrement [18]. Last, Oceania exhibits increasing TBLC due to the increase of higher SP rates within the countries such as Australia and New Zealand, mainly where regions too contribute to show that TBLC becomes significant in countries at a low or higher level [19–21].

Moreover, the correlation between SP and TBLC shows a strong association. Although smoking is the modifiable significant primary risk factor which causes LC, other contributing factors such as occupational exposure, air pollution, second-hand smoke, occupational exposure to asbestos and radon, genetic predisposition and environmental factors like air pollution also contribute towards the development of LC mortality rates [22–24].

SPs effect on TBLC is critical; and also, the effect that the HAQ index exerted towards the relationship of SP on TBLC, tends to show variations due to the changes occurred. This study aims to investigate the interaction effect of SP and the HAQ index on TBLC mortality rates across continents over a 30-year period. Therefore, this study aims to contribute mainly to such body of knowledge through five prongs. First, this study identifies the correlation between SP and TBLC in a continental aspect and reinforces the mandatory need for tobacco control and anti-smoking campaigns. This also considers the effect with the healthcare access as that also directly or indirectly results in the change of TBLC mortality rates occurred due to SP and it also should be considered in implementing such measure.

Second, the importance of improving the healthcare infrastructure for better treatment against TBLC can be imposed by assessing the HAQ index against TBLC deaths. Moreover, future research studies can consider the Gross Domestic Product with the income levels, to uplift the poor and limited HAQ levels specifically to demise the TBLC death rates.

Third, this research assists to examine the influence of SP and healthcare access towards TBLC at a continental level, where limited continents with healthcare can be focused, and further investments can be made. Therefore, the quality of healthcare can be maximised so that the death rates due to TBLC can be minimised gradually.

Fourth, an analysis of death rates across 30 years guides policymakers in enhancing and establishing smoking cessation initiatives and TBLC cessation programs. This helps the population get rid of tobacco smoking while enhancing their health status. Finally, the study’s findings can be utilised in evidence-based policy recommendations for reducing SP and increasing the HAQ index to tackle TBLC death rates effectively.

## Literature review

Mortality rates due to TBLC have been an evolving significant health concern where SP and healthcare access impose a crucial influence within a continental aspect [25, 26]. Analysing and synthesising past literary appreciations at a continental level has been undertaken to prove the strong association between the variables considered.

Several researchers have investigated this intricate relationship in scrutinising the effect of SP and HAQ index on TBLC mortality rates globally. SP and its association with tracheal bronchitis reveal a strong and positive correlation between the considered variables by analysing data from multiple countries, enhancing the robustness of the key findings through the research conducted [27]. Moreover, the combined impact of SP and TBLC was analysed and depicted that regions bearing a higher SP with limited healthcare access are exposed to significantly increasing TBLC mortality rates and has emphasised the fact that implementing tobacco-free policies and healthcare infrastructure to combat TBLC [28]. Additionally, a study on the relationship between the HAQ index and LC deaths highlighted that developed healthcare access was associated with lower LC rates varying across countries [29]. These statistical studies underscore the urge to uplift public health strategies and mitigate SP to terminate the risk of TBLC deaths globally through advanced pre-emptive measures.

In addition, the dual continents Asia and Africa also visualise the relationship between SP and healthcare access affecting the LC death rates. Some studies proved that the disease prevention rate could be minimised to some extent through a systematic approach to improving screening and health follow-up [30–32]. Another perspective on Asia depicts that increasing tobacco taxes significantly decreases SP and attributable death rates [33, 34]. Comparatively, these studies used primary data investigations for a limited time frame and focused only on specific continents, expecting it to be done from a global perspective. At the same time, all three aspects, SP, HAQ index and TBLC death rates, haven’t been directly considered in these studies.

A study focussing on the European continent has illuminated that a positive association lies between SP and TBLC. At the same time, the HAQ index moderates this strong association by implying variations and lower exacerbating the effect of smoking on TBLC [14]. Furthermore, some studies depict through a comprehensive analysis across European countries, that healthcare access shows a link with LC deaths, specifically in countries with an increasing rate of SP. The regional disparities in healthcare and its impact on TBLC demonstrated that higher healthcare infrastructure relies on lower mortality rates from TBLC, highlighting the need for healthcare across the continent [35–38]. Overall, the unification of these studies adds significant perceptions towards the interplay between the variables addressed and lays the groundwork for policy formulation and public health concerns.

Likewise, this relationship has been previously explored in various aspects within the American continent. In detailing the lung-related cancer deaths, it was evident that they were caused due to the use of alcohol and tobacco consumption, mainly Silversmith [39–41]. Further, a previously mentioned study indicates that females (7%) were more into LC deaths than males in more developed countries. Conversely, these studies employ primary analytical techniques concerning only limited variables and study frames. 

Various researchers have considered the relationship between the increase of LC and SP in gender-wise categorisation. The countries which represent the Oceanian continent too show a higher bias of LC in men in most cases when compared to females but varies, according to the studies [26, 42–45]. Some of these studies employed meta-analysis pooled results, linear and Poisson log-regression models in analysis with a limited number of years considered. Similarly, most of the studies provide policy implications, while some studies suggest that cancer control programs and higher standard health tests are cost-effective within the countries [46–48]. Therefore, the studies are more aligned with the increase in LC deaths when measuring the effect of healthcare and SP in continental aspects as well as globally.

### Theoretical foundation

The complex relationship between SP and HAQ index on the TBLC death rates rests on the foundation of the socio-ecological model, which helps to understand the interplay between individual, interpersonal, community and societal factors which influence SP, HAQ index in concern towards the TBLC death rates. It helps to investigate the multi-dimensional attributes of this relationship. At the individual level, SP can be influenced through attitudes, beliefs, knowledge, and personal choices. At the interpersonal level, peer influences, social norms, and the family will affect the SPs behaviors and habits. Tobacco prevalence policies and health campaigns can be considered in the community stage. Finally, government-embedded policies, economic disparities and healthcare facilities fall under the societal factors. This model effectively helps design public policies and health facilities regarding TBLC deaths [49].

Conversely, Health Belief Model also can postulate how a person’s beliefs about the susceptibility towards TBLC deaths influence their smoking and Healthcare access-related decisions [50]. Moreover, Social Cognitive Theory can be utilised in this study to observe the smoking behaviours and the expected outcomes of healthcare influencing SP and healthcare access among individuals [51, 52]. Ecological Systems Theory can be applied to understand how factors such as family, community, society, and workplace contribute towards HAQ and smoking behaviours [53, 54]. Lastly, through the Behavioural Economics Model, the factors which influence the preferences of cessation programs and healthcare infrastructure can be identified [55, 56].

## Method

This study explores the main effect of SP and HAQ index towards TBLC through a continental approach for 204 countries between 1990 and 2019, covering a 30-year time frame. The empirical analysis relies on data obtained from the Global Burden of Disease (GBD) database [57] for SP (converted to per 100,000 people) and the HAQ index [58]. SP, typically reported as a percentage was converted to per 100,000 people to harmonise its measurement with the dependent variable which is conventionally expressed in the same unit, thereby enabling comparability in analysis and visualisation. Moreover, the secondary data for TBLC (per 100,000 people) were retrieved from the Our World in Data database [59]. The categorization thresholds for SP and HAQ were determined based on the distribution of the dataset and aligned with public health benchmarks reported in global Institute of Health Metrics and Evaluation to ensure meaningful stratification. Countries with missing data were excluded from the analysis to preserve data quality. The data file employed for the study is presented in the S1 Appendix.

The dataset was structured as a balanced panel spanning 30 years across the included continents to analyse the interaction of SP and HAQ index on TBLC, and a two-way analysis of variance (ANOVA) was regressed, including SP and HAQ index as covariates to assess the influence of SP and healthcare access on LC mortality rates [60–62]. This method was selected as it is robust for exploring interaction effects and allows for testing differences across multiple categorical groupings while accounting for potential confounding due to combined effects. The independent variables were categorised into four levels each as Low SP (0–14.99), Moderate SP (15–23.99), High SP (24–30.99), and Critical SP (31–100) for the SP variable, and Poor HAQ (0–39.99), Limited HAQ (40–54.99), Adequate HAQ (55–69.99), and Optimal HAQ (70–100) in consideration to the HAQ index and 0.05 significance level was considered in analysing the data for the study. Continental-wise two-way ANOVA results are presented in the S2 Appendix. For further clarifications the continental wise two-way mean ANOVA results and the interaction plots for the SP levels are attached under S3 Appendix and S4 Appendix.

To ensure the validity and robustness of the panel estimations, a series of specification and sensitivity tests were conducted. The F-test confirmed the relevance of the Fixed Effects (FE) model over pooled OLS, while the Breusch Pagan Lagrange Multiplier (LM) test supported the application of Random Effects (RE) models over pooled OLS. Subsequently, the Hausman test consistently indicated the appropriateness of the FE estimator across all continents, suggesting that unobserved heterogeneity is correlated with the regressors. In addition, all estimations were re-specified with heteroskedasticity robust standard errors using the Modified Wald test for groupwise heteroskedasticity in the FE regression model to account for variance heterogeneity across continents. The detailed test results and FE estimations are provided in the S5 Appendix, and they demonstrate that the main findings remain stable under these alternative specifications. Further, Stata was used as the analytical tool, and R Studio was utilised as the graphical tool.

## Results

This section provides insights into the significant findings and the discussions brought out through them within the continents. Further, a bar plot visualising the relationship between the dependent and categorical variables graphically illustrates the behaviour of the means and standard deviations.

**Fig. 1:**
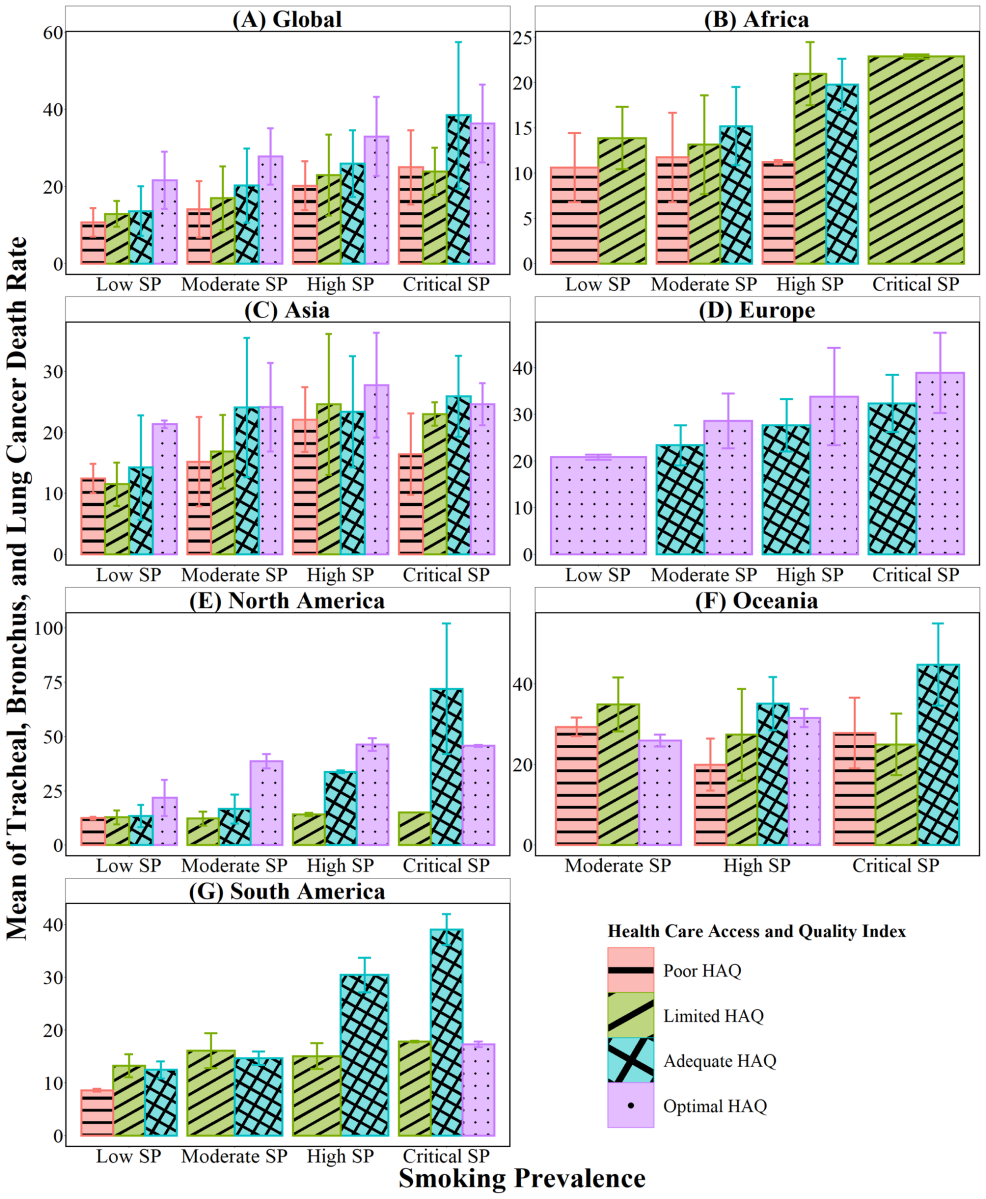
Bar plot for the two factors SP and HAQ index continent-wise. Source: Authors’ illustration based on data

The bar plots indicating the mean and standard deviation values of the levels of the HAQ index for the considered SP levels globally and continent wise is depicted in Fig. 1. According to Fig. 1 (A), it was discovered that average TBLC deaths were the highest in countries with Critical SP and Adequate HAQ levels and lowest in countries with Low SP and Poor HAQ levels worldwide. Likewise, for the African region in Fig. 1 (B), countries with critical SP and Limited HAQ levels indicated the most TBLC deaths. The Asian countries with moderate SP levels showed similar TBLC mortality rates for adequate and optimal HAQ, concerning Fig. 1 (C). In the European region, with the countries falling under adequate and optimal HAQ levels, it was observed in Fig. 1 (D) that TBLC deaths were comparatively higher for optimal HAQ for all the levels of SP. For the North American, Oceanian, and South American regions portrayed in Fig. 1 (E), Fig. 1 (F), and Fig. 1 (G), respectively, countries with adequate HAQ and critical SP had the highest TBLC death rate.

**Fig. 2:**
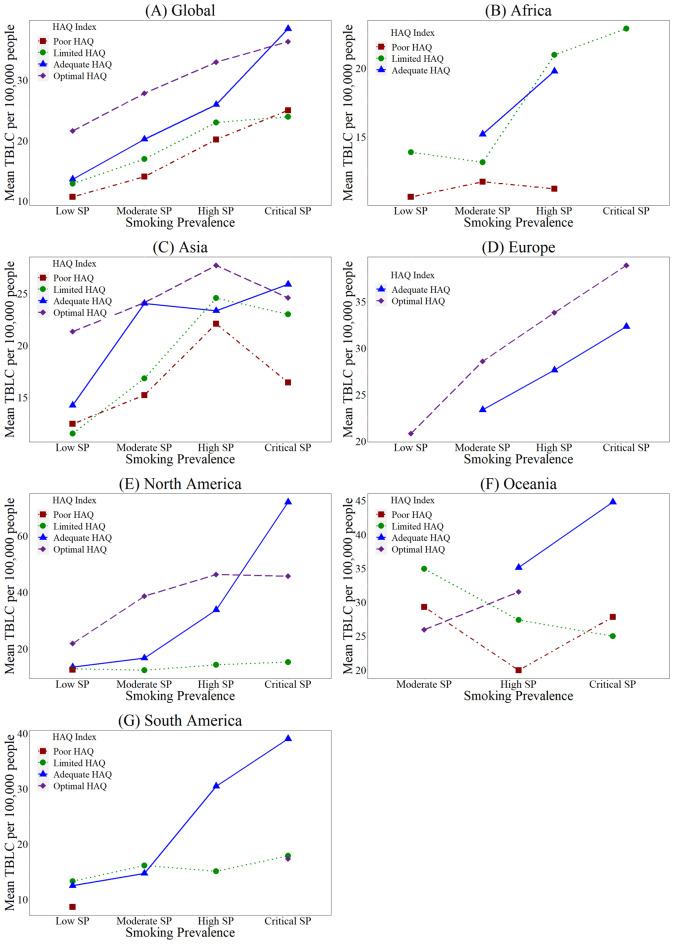
Interaction plots for the HAQ index levels. Source: Authors’ illustration based on data

The influence of SP and HAQ index on the TBLC was assessed by implementing a two-way ANOVA globally and continent-wise. The global results depict statistically significant main effects for SP and HAQ index and their interaction at a 1% significance level. The main impact for SP shows [F (3,6104) = 573.05, *p* < 0.0000] which portrays a significant difference between four categories: low SP (*M* = 12.21, SD = 4.84), moderate SP (*M* = 18.95, SD = 9.52), high SP (*M* = 27.35, SD = 10.49) and critical SP (*M* = 33.78, SD = 14.02). The main effect of HAQ index depicts [F (3,6104) = 359.65, *p* < 0.0000] portraying a significantly lower TBLC value in poor HAQ (*M* = 14.02, SD = 7.73) when compared with the other categories, limited HAQ (*M* = 16.85, SD = 8.19), Adequate HAQ (*M* = 25.05, SD = 14.55) and Optimal HAQ (*M* = 32.68, SD = 10.36). There was also a significant interaction between SP and HAQ index when the effect on TBLC is considered at [F (9,6104) = 29.27, *p* < 0.0000], which signifies that the relationship between SP and TBLC also depends on the HAQ index. Further, through the interaction plots depicted in Fig. 2, the results can be more stressed as Fig. 2 (A) portrays that the effect between Adequate and optimal HAQ is more potent in critical SP. At the same time, limited and poor HAQ shows a weaker intersection.

Revolving around the African continent, the results show that both SP and HAQ indices have significant main effects, SP [(F = 18.20, p-value = 0.0000)] and HAQ [(F = 49.05, p-value = 0.0000)] where both variables significantly impact on TBLC death rates. The interaction effect (F = 11.33, p-value = 0.0000) also confirms a joint influence on TBLC mortality. Figure 2 (B) depicts that interaction between adequate and limited HAQ can be seen at moderate SP, and on the other hand, the effect of the HAQ index is consistent across the four SP levels.

Further, the Asian continent portrays significant main effects, SP [(F = 36.93, p-value = 0.0000)] and HAQ [(F = 39.61, p-value = 0.0000)], where each distinct level of SP and HAQ index influences the TBLC death rates significantly. The interaction between SP and HAQ index (F = 7.32) statistically signifies the joint effect towards the TBLC death rates at a 1% significance level. As further clarified through Fig. 2 (C), a substantial impact on adequate and optimal HAQ is portrayed between SPs Low and critical levels. At the same time, limited HAQ shows a strong interaction with high SP and a weak one with low SP. Further, poor and limited HAQ shows a soft effect with low SP. According to the analysis of the Fig. 2 (D) reveals that SP and HAQ show statistically significant main where each variable has an individual impact towards TBLC death rates. On the contrary, the interaction effect of SP and HAQ indicates that the prevalence of smoking and the HAQ index does not explain the variance of TBLC deaths significantly, although they show an impact individually.

The American continent, which comprises North and South American regions, shows that the SP and HAQ index levels substantially impact the TBLC mortality rates when considering the main effects. Moreover, the interaction effects show significance which prevails that the combination of SP and HAQ index highly contributes towards the deaths caused due to TBLC. Furthermore, Fig. 2 (E) portrays an interaction in high SP between adequate and optimal HAQ. At the same time, a lesser overlap can be seen, representing a weak effect of adequate and limited HAQ on low SP. Then Fig. 2 (F), evident a weak effect between adequate and limited HAQ within the moderate level of SP.

Moreover, the result of the Oceanian continent predicts that the main effects become significant, SP (F = 14.21) and HAQ (F = 73.16) at a 1% significance level where countries depict distinct SP and HAQ level outcomes showing an influence towards the TBLC death values. Further, the interaction of SP and HAQ index, [(F = 20.72, p-value = 0.000)], shows a notable combination impact towards the mortality rates due to TBLC as this interplay creates unique conditions affecting the dependent variable. Finally, Fig. 2 (G) outliers the effect of optimal HAQ with both poor and limited HAQ in moderate and high SP levels, while adequate HAQ shows no interaction towards the other levels.

## Discussion

Further, moving on, in this study, it is evident that there is a significant interaction between the four SP levels and HAQ levels, with differences between each group towards the mortality rate occurring due to TBLC overall globally and continental-wise. At a global level, better health outcomes were represented where a higher HAQ index with lower SP through the optimal level. Conversely, poor health factors were identified in the poor category, where it resulted in deaths caused due to TBLC proportionally. Several previous studies have reported that smoking is associated with a higher proportion of LC-related deaths [63–66]. In one case-control study, it is mentioned that despite the histologic type of LC and the metric of smoking which was considered, high smoking levels have a higher risk of death irrespective towards gender [67, 68]. Nevertheless, previous studies were mainly population-based cohort studies and case-control studies, which implied a comparatively small population [66, 69]. On the contrary, this study implies data from about 30 years investigating the relations between health care and SP towards TBLC-related deaths. Contrastingly, an unexpected finding of the study states that TBLC rates were lowest in regions with Low SP and Poor HAQ and highest in regions with Critical SP and Adequate HAQ. This counterintuitive pattern should be interpreted with caution, as it may reflect unmeasured confounding factors such as differences in reporting quality, competing mortality risks, and demographic age structures. These issues highlight the importance of more detailed future research to examine such patterns with additional controls and alternative modelling approaches.

The African region depicts notable variations within the HAQ index levels, where the results show that countries with a low SP level with an adequate HAQ intend to have good health access compared to those with a higher SP and poor HAQ index. Some studies show that despite the racial disparities, gender, and other causes of LC deaths tobacco smoking accelerates the risk of TBLC deaths by a higher proportion [25, 70]. On the other hand, it is encouraged to increase health prevention efforts as most African countries fall under the low-income stratum [71]. Therefore, this can provide insights towards the governments where health access lies and the effort that should be enhanced to strengthen the immunity and healthcare systems to limit TBLC deaths.

Revolving around the results shows that areas with higher HAQ index and lower SP have better health effects. It emphasises the importance of healthcare access and the reduction of SP to enhance health by limiting TBLC deaths within the Asian region. Further, smoking with other combinative factors also shows an inclination to TBLC deaths [72–75]. Conversely, countries should invest more in healthcare facilities on cancer screening to reduce deaths due to TBLC.

On the contrary, from the results of this research, which see no interaction between healthcare and SP towards TBLC deaths in Europe, a study conducted considering Switzerland has shown that 6–15% LC reduction overshadows LC screenings which depict the advancement of healthcare access as a 2–6% increasing rate is also identified due to SP [76]. Further studies done revolving around Scotland also depicts that there are urban influences on the incidence of lung cancer that are not totally explained by smoking behaviours [77, 78]. Unfortunately, this may be due to the lack of data in some categories of the categorical variables.

Through the results of this study, it was evident that pertaining to the American continent, North America demonstrates that areas with lower SP overshows better health outcomes where the HAQ index seems to be high and emphasises that SP reduction limits deaths caused due to TBLC. It is concordant with previous research that screening of early LC induced due to SP and other factors helps in the reduction of death rates and signifies updated healthcare access [79–83]. Another study demonstrated that the TBLC mortality outcomes showed variations due to facts like tobacco control and access to healthcare [84]. A cohort analysis using a log-linear poison model proved that 85% of TBLC-related deaths are attributed to SP [85]. Hence, unmeasured confounding variables could also result in TBLC deaths, but the healthcare system and policy implications against the smoking hazard can play a significant role in TBLC deaths. In contrast, South America needs a deeper investigation of healthcare access as the region shows mixed patterns of SP and HAQ index and influences in TBLC rates accordingly. Obscuration of the relationship between prevalence factors and HAQ index levels results from the limitation of the data in some categories. A population-based study showed that most countries’ healthcare systems do not support the cessation of SP, which causes a higher burden in TBLC death rates [86]. In addition, a recent study depicts the global SP patterns by linking it with healthcare access and LC mortality. This emphasises how disparities in healthcare systems amplify the adverse effects of tobacco use towards the population. This study builds on this evidence by examining in a continental approach exploring the effects of SP and HQA [87]. However, with the vast development of healthcare facilities, the population get immense opportunities to maintain good health. At the same time, the government invests in policy implications where a notable amount of people chooses to quit smoking.

Diverse SP and HAQ index patterns portray the complex integration between healthcare access and SP towards TBLC deaths within Oceania. Unfortunately, the lack of data towards some categories like “Poor” limits the understanding of the health disparities, whereas it can be interpreted as the continent having good health facilities comparatively. Dual studies portray the decline of mortality rates induced by SP, while a study demonstrated that about 20% of LC deaths were averted due to tobacco control until 2015 [88, 89]. Another study using the macrosimulation stage shift model casts a high disease burden from LC, where the healthcare system perspective should be cost-effective [69]. This obscure relationship helps the government implement laws related to smoking cessation and invest more in uplifting immunity and healthcare.

Finally, this research acknowledges its limitations, including the potential confounding variables and unmeasured factors such as socioeconomic status, cultural norms and individual health behaviours, which influence this relationship and might impact the results and may require future research. At the same time, data granularity can introduce challenges when aggregating or comparing results globally, potentially leading to under- or overestimation of TBLC mortality rates in certain regions. Further, the future studies could explore the dynamic temporal trends for additional insights using this extended dataset. Moving forward, there may be limitations in the HAQ index as it relies on specific metrics; ecological fallacy in aggregating data continental-wise might hold individual relations at a group level. Moreover, this study uses population-level data, so the findings may not apply to individuals, and combining 30 years of data could overlook changes over time. Further, data regarding SP and HAQ index might rely on self-report bias. On the other hand, this study does not incorporate lag structures to reflect the latency between smoking and the lung cancer outcomes, therefore this can be addressed in a future research to capture the delayed epidemiological effects more precisely. Lastly, this study uses categorical groupings of SP and HAQ and employed two-way ANOVA to illustrate broad associations. While this approach was suited to the exploratory scope and heterogeneous nature of the data, it does not fully exploit the advantages of panel econometric models. Future research should extend this work by modelling SP and HAQ as continuous variables with interaction terms, incorporating continent and year fixed effects, and adjusting for additional covariates such as age structure, economic indicators, and alternative cut-points to test robustness.

## Conclusion

This study concisely evaluates the complex relationship between SP and HAQ index on TBLC death rates by providing acumen information. The findings underscore the importance of addressing this relationship by categorising healthcare access and SP levels to identify the distinct patterns across the continents, highlighting the need to promote better health facilities and tailoring interventions towards the regional disparities. This study emphasises the crucial need for public and tobacco control policies to enhance healthcare access by promoting better LC-related health worldwide.

Overall, this study contributes towards the growing literature on global health and smoking cessation, prioritising healthcare access to reduce TBLC death rates. Therefore, policymakers and health professionals can implement interventions that improve TBLCs worldwide well-being, along with a healthier future with minimised SP and increased health with limited TBLC-related people.

### Policy implications

This studys’ findings suggest important policy implications for public health, particularly in regions where critical SP and poor HAQ occur and are associated with elevated TBLC mortality. The observed correlations between SP, HAQ index and TBLC death rates highlight the potential value of region-specific interventions that address both tobacco control and healthcare system strengthening. Policymakers could consider prioritising in enhancing healthcare systems in countries with poor or limited HAQ, focusing on improving early diagnosis and treatment for TBLC and implementing robust smoking cessation programs, especially in regions with high SP such as Asia, Africa and TBLC is disproportionally high. Moreover, regulating tobacco control legislation, promoting anti-smoking initiatives, and organising awareness campaigns and programs on smoking-related health effects will encourage individuals to quit smoking and prevent new smokers from starting over the habit crucially in continents classified as having critical SP levels in the study. Concurrently, efforts to improve healthcare access and quality should aim on reducing medical service obstacles, ensuring affordability, and implementing infrastructure simultaneously.

The policies will help in positive health outcomes, specifically in areas with poor health access and critical SP level. Moreover, collaboration between health agencies, policymakers and community organisations is essential to establishing approaches that tackle the SP and HAQ indexs’ impact on TBLC death rates. Finally, evidence-based policies can help regulations across the continents improve their well-being by reducing the burden of TBLC-related deaths and controlling the smoking-related prevalence.

## Electronic supplementary material

Below is the link to the electronic supplementary material.

Supplementary Material 1: S1 Appendix. Data file

Supplementary Material 2: S2 Appendix. Continental wise two-way ANOVA results

Supplementary Material 3: S3 Appendix. Continental wise two-way mean ANOVA results

Supplementary Material 4: S4 Appendix. Interaction plots for the SP levels

Supplementary Material 5: S5 Appendix. Continental Panel Regression Results using Fixed Effect Estimator

## Data Availability

All data generated or analysed during this study are included in this published article and its supplementary information files.

## Abbreviations

ANOVA: Analysis of Variance
FE: Fixed Effect
GBD: Global Burden of Disease
HAQ: Healthcare Access and Quality
LC: Lung Cancer
RE: Random Effect
SP: Smoking Prevalence
TBLC: Trachea, Bronchus, and Lung Cancer
