# Supplementary material for: Global nexus of smoking prevalence, healthcare quality and respiratory cancer mortality: a cross-continental study
Source: BMC Health Serv Res. 2025 Oct 6;25:1307. doi: 10.1186/s12913-025-13508-9 (PMC12502336; doi:10.1186/s12913-025-13508-9)
Supplement: Supplementary file 2 — Supplementary Material 2: S2 Appendix. Continental wise two-way ANOVA results [file 12913_2025_13508_MOESM2_ESM.docx]

**S2 Appendix: Continental-wise two-way ANOVA results**

| **Variable** | **Sum of squares** | **df** | **Mean Square** | **F** | **p-value** |
| --- | --- | --- | --- | --- | --- |
| Model | 532354.85 | 15 | 35490.324 | 464.90 | 0.0000 |
| SP | 131238.35 | 3 | 43746.116 | 573.05 | 0.0000 |
| HAQ Index | 82367.737 | 3 | 27455.912 | 359.65 | 0.0000 |
| SP#HAQ | 20108.648 | 9 | 2234.294 | 29.27 | 0.0000 |
| Residual | 465977.81 | 6,104 | 76.340 |  |  |
| Total | 998332.67 | 6,119 | 163.153 |  |  |
| No. of Observations | 6,120 | | | | |
| R-Square | 0.5332 | | | | |

ST2.1: Global two-way ANOVA results

ST2.2: Two-way ANOVA results for the African Continent

| **Variable** | **Sum of squares** | **df** | **Mean Square** | **F** | **p-value** |
| --- | --- | --- | --- | --- | --- |
| Model | 8411.826 | 8 | 1051.478 | 61.96 | 0.0000 |
| SP | 926.596 | 3 | 308.865 | 18.20 | 0.0000 |
| HAQ Index | 1664.756 | 2 | 832.378 | 49.05 | 0.0000 |
| SP#HAQ | 576.951 | 3 | 192.317 | 11.33 | 0.0000 |
| Residual | 27340.121 | 1,611 | 16.971 |  |  |
| Corrected total | 35751.947 | 1,619 | 22.083 |  |  |
| No. of Observations | 1,620 | | | | |
| R-square | 0.235 | | | | |

ST2 3: Two-way ANOVA results for the Asian Continent

| **Variable** | **Sum of squares** | **df** | **Mean Square** | **F** | **p-value** |
| --- | --- | --- | --- | --- | --- |
| Model | 35260.669 | 15 | 2350.711 | 35.24 | 0.0000 |
| SP | 7391.131 | 3 | 2463.710 | 36.93 | 0.0000 |
| HAQ Index | 5925.331 | 3 | 1975.110 | 29.61 | 0.0000 |
| SP#HAQ | 4395.974 | 9 | 488.442 | 7.32 | 0.0000 |
| Residual | 96994.673 | 1,454 | 66.709 |  |  |
| Corrected total | 132255.34 | 1,469 | 90.031 |  |  |
| No. of Observations | 1,470 | | | | |
| R-square | 0.267 | | | | |

ST2.4: Two-way ANOVA results for the European Continent

| **Variable** | **Sum of squares** | **df** | **Mean Square** | **F** | **p-value** |
| --- | --- | --- | --- | --- | --- |
| Model | 23597.143 | 6 | 3932.857 | 56.33 | 0.0000 |
| SP | 10976.603 | 3 | 3658.868 | 52.41 | 0.0000 |
| HAQ Index | 4598.126 | 1 | 4598.126 | 65.86 | 0.0000 |
| SP#HAQ | 35.188 | 2 | 17.594 | 0.25 | 0.7773 |
| Residual | 87483.094 | 1,253 | 69.819 |  |  |
| Corrected total | 111080.24 | 1,259 | 88.230 |  |  |
| No. of Observations | 1,260 | | | | |
| R-square | 0.212 | | | | |

ST2.5: Two-way ANOVA results for the North American Continent

| **Variable** | **Sum of squares** | **df** | **Mean Square** | **F** | **p-value** |
| --- | --- | --- | --- | --- | --- |
| Model | 186855.09 | 12 | 15571.258 | 224.70 | 0.0000 |
| SP | 15438.264 | 3 | 5146.088 | 74.26 | 0.0000 |
| HAQ Index | 7195.426 | 3 | 2398.475 | 34.61 | 0.0000 |
| SP#HAQ | 16518.107 | 6 | 2753.018 | 39.73 | 0.0000 |
| Residual | 87483.094 | 797 | 69.297 |  |  |
| Corrected total | 55229.778 | 809 | 299.240 |  |  |
| No. of Observations | 810 | | | | |
| R-square | 0.772 | | | | |

ST2.6: Two-way ANOVA results for the South American Continent

| **Variable** | **Sum of squares** | **df** | **Mean Square** | **F** | **p-value** |
| --- | --- | --- | --- | --- | --- |
| Model | 23597.143 | 9 | 2215.895 | 369.04 | 0.0000 |
| SP | 10976.603 | 3 | 1947.523 | 324.35 | 0.0000 |
| HAQ Index | 4598.126 | 3 | 2183.870 | 363.71 | 0.0000 |
| SP#HAQ | 35.188 | 3 | 1472.998 | 245.32 | 0.0000 |
| Residual | 87483.094 | 350 | 6.004 |  |  |
| Corrected total | 111080.24 | 359 | 61.406 |  |  |
| No. of Observations | 360 | | | | |
| R-square | 0.905 | | | | |

ST2.7: Two-way ANOVA results for the Oceanian Continent

| **Variable** | **Sum of squares** | **df** | **Mean Square** | **F** | **p-value** |
| --- | --- | --- | --- | --- | --- |
| Model | 19347.915 | 9 | 2149.768 | 33.75 | 0.0000 |
| SP | 1809.563 | 2 | 904.782 | 14.21 | 0.0000 |
| HAQ Index | 13977.82 | 3 | 4659.273 | 73.16 | 0.0000 |
| SP#HAQ | 5278.929 | 4 | 1319.732 | 20.72 | 0.0000 |
| Residual | 37577.195 | 590 | 63.690 |  |  |
| Corrected total | 111080.24 | 599 | 95.034 |  |  |
| No. of Observations | 360 | | | | |
| R-square | 0.905 | | | | |
