# Supplementary material for: Global nexus of smoking prevalence, healthcare quality and respiratory cancer mortality: a cross-continental study
Source: BMC Health Serv Res. 2025 Oct 6;25:1307. doi: 10.1186/s12913-025-13508-9 (PMC12502336; doi:10.1186/s12913-025-13508-9)
Supplement: Supplementary file 3 — Supplementary Material 3: S3 Appendix. Continental wise two-way mean ANOVA results [file 12913_2025_13508_MOESM3_ESM.docx]

**S3 Appendix: Continental wise two-way mean ANOVA results**

ST3.1: Mean values for SP and HAQ index at a Global level

|  | **HAQ Index** | | | |
| --- | --- | --- | --- | --- |
| **SP** | Poor | Limited | Adequate | Optimal |
| Low | 10.683 + 3.699 | 12.882 + 3.335 | 13.609 + 6.460 | 21.593 + 7.431 |
| Moderate | 14.055 + 7.308 | 16.932 + 8.282 | 20.222 + 9.604 | 27.809 + 7.300 |
| High | 20.174 + 6.336 | 22.978 + 10.487 | 25.939 + 8.661 | 32.974 + 10.273 |
| Critical | 24.972 + 9.650 | 23.911 + 6.114 | 38.476 + 19.004 | 36.361 + 10.103 |

ST3.2: Mean values for SP and HAQ index for the African Continent

|  | **HAQ Index** | | | |
| --- | --- | --- | --- | --- |
| **SP** | Poor | Limited | Adequate | Optimal |
| Low | 10.616 + 3.813 | 13.868 + 3.441 | - | - |
| Moderate | 11.726 + 4.935 | 13.130 + 5.443 | 15.181 + 4.300 | - |
| High | 11.197 + 0.231 | 20.970 + 3.501 | 19.781 + 2.841 | - |
| Critical | - | 22.87 + 0.240 | - | - |

ST3.3: Mean values for SP and HAQ index for the Asian Continent

|  | **HAQ Index** | | | |
| --- | --- | --- | --- | --- |
| **SP** | Poor | Limited | Adequate | Optimal |
| Low | 12.441 + 2.422 | 11.496 + 3.526 | 14.224 + 8.571 | 21.388 + 0.614 |
| Moderate | 15.193 + 7.333 | 16.824 + 6.034 | 24.053 + 11.402 | 24.115 + 7.246 |
| High | 22.085 + 5.312 | 24.579 + 11.537 | 23.348 + 9.110 | 27.712 + 8.608 |
| Critical | 16.424 + 6.687 | 22.999 + 1.940 | 25.888 + 6.619 | 24.591 + 3.438 |

ST3.4: Mean values for SP and HAQ index for the European Continent

|  | **HAQ Index** | | | |
| --- | --- | --- | --- | --- |
| **SP** | Poor | Limited | Adequate | Optimal |
| Low | - | - | - | 20.816 + 0.565 |
| Moderate | - | - | 23.364 + 4.313 | 28.573 + 5.902 |
| High | - | - | 27.634 + 5.615 | 33.790 + 10.429 |
| Critical | - | - | 32.306 + 6.130 | 38.865 + 8.599 |

ST3.5: Mean values for SP and HAQ index for the North American Continent

|  | **HAQ Index** | | | |
| --- | --- | --- | --- | --- |
| **SP** | Poor | Limited | Adequate | Optimal |
| Low | 12.436 + 0.642 | 12.788 + 3.253 | 13.427 + 5.162 | 21.743 + 8.310 |
| Moderate | - | 12.268 + 3.182 | 16.622 + 6.737 | 38.597 + 3.347 |
| High | - | 14.149 + 0.780 | 33.743 + 0.639 | 46.325 + 2.928 |
| Critical | - | 15.11 + NA | 71.993 + 30.089 | 45.684 + 0.401 |

ST3.6: Mean values for SP and HAQ index for the Oceanian Continent

|  | **HAQ Index** | | | |
| --- | --- | --- | --- | --- |
| **SP** | Poor | Limited | Adequate | Optimal |
| Low | - | - | - | - |
| Moderate | 29.279 + 2.323 | 34.900 + 6.719 | - | 25.949 + 1.479 |
| High | 19.949 + 6.426 | 27.372 + 11.383 | 35.105 + 6.599 | 31.522 + 2.225 |
| Critical | 27.822 + 8.772 | 24.976 + 7.603 | 44.756 + 10.203 | - |

ST3.7: Mean values for SP and HAQ index for the South American Continent

|  | **HAQ Index** | | | |
| --- | --- | --- | --- | --- |
| **SP** | Poor | Limited | Adequate | Optimal |
| Low | 8.626 + 0.258 | 13.268 + 2.192 | 12.473 + 1.603 | - |
| Moderate | - | 16.100 + 3.298 | 14.701 + 1.292 | - |
| High | - | 15.092 + 2.447 | 30.457 + 3.270 | - |
| Critical | - | 17.853 + 0.163 | 39.016 + 2.940 | 17.343 + 0.536 |
