# Supplementary material for: Global nexus of smoking prevalence, healthcare quality and respiratory cancer mortality: a cross-continental study
Source: BMC Health Serv Res. 2025 Oct 6;25:1307. doi: 10.1186/s12913-025-13508-9 (PMC12502336; doi:10.1186/s12913-025-13508-9)
Supplement: Supplementary file 4 — Supplementary Material 4: S4 Appendix. Interaction plots for the SP levels [file 12913_2025_13508_MOESM4_ESM.docx]

**S4 Appendix: Interaction plots for the SP levels**

SF 4.1: Interaction plots for the SP levels.


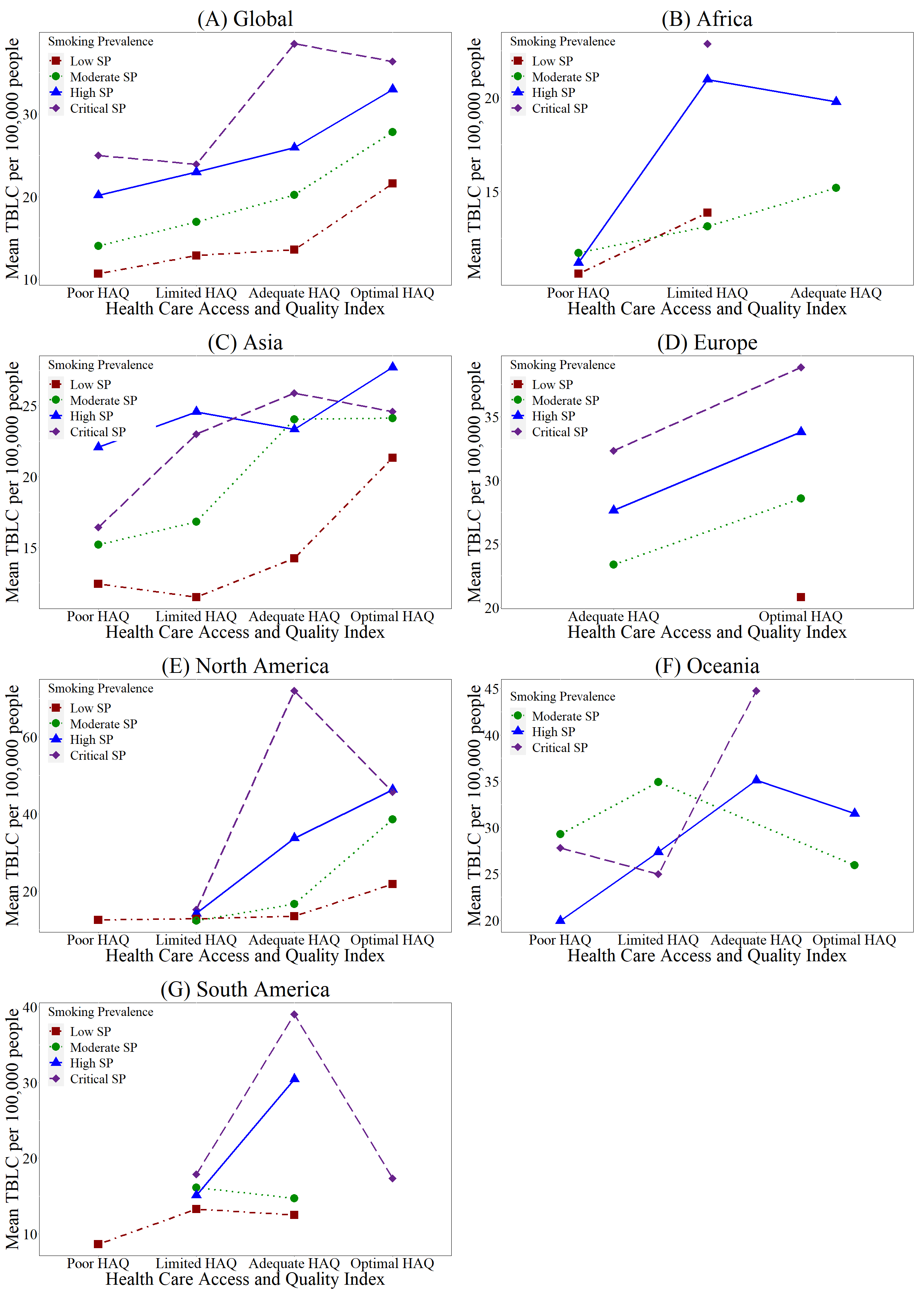


Source: Authors’ illustration based on data
