# Supplementary material for: Global nexus of smoking prevalence, healthcare quality and respiratory cancer mortality: a cross-continental study
Source: BMC Health Serv Res. 2025 Oct 6;25:1307. doi: 10.1186/s12913-025-13508-9 (PMC12502336; doi:10.1186/s12913-025-13508-9)
Supplement: Supplementary file 5 — Supplementary Material 5: S5 Appendix. Continental Panel Regression Results using Fixed Effect Estimator [file 12913_2025_13508_MOESM5_ESM.docx]

**S5 Appendix: Continental Panel Regression Results using Fixed Effect Estimator**

|  | **African** | **Asian** | **European** | **North American** | **South American** | **Oceanian** |
| --- | --- | --- | --- | --- | --- | --- |
| **Variable** | **FE** | | | | | |
| TBLC | -0.0034** | 0.1291*** | 0.0346** | 0.0509* | 0.0137*** | 0.0489** |
| Constant | 12.4009*** | 17.879*** | 23.4460*** | 11.8157*** | 14.5309* | 13.8333*** |
| R^2^ | 0.0867 | 0.1076 | 0.1474 | 0.6959 | 0.3534 | 0.0003 |
| F-Test | 688.59*** | 2.03*** | 9.92** | 6.94* | 9.08*** | 227.53** |
| LM Test | 21202.26*** | 16930.41*** | 12320.86*** | 8812.28*** | 4268.39*** | 8057.48*** |
| Hausman Test | 8.29*** | 3.57*** | 2.25* | 1015.03*** | 16.89*** | 4.12** |
| N | 1620 | 1470 | 1260 | 810 | 360 | 600 |

Note: The asterisks, *, ** and *** indicate 10%, 5% and 1% significance level, respectively. FE represent the Fixed Effect, and N shows the number of observations. The hypothesis for F-test is Ho: Accepting POLS and Ha: Accepting FE Model, the hypothesis for the Breusch-Pagan test is Ho: Accepting POLS and Ha: Accepting RE Model and the hypothesis for the Hausman test are Ho: Accepting RE Model and Ha: Accepting FE Model.
